# Exploring the Potential of Seaweed Derivatives for the Development of Biodegradable Plastics: A Comparative Study

**DOI:** 10.3390/polym15132884

**Published:** 2023-06-29

**Authors:** Wan Amnin Wan Yahaya, Nurul Aini Mohd Azman, Fatmawati Adam, Sarmilaah Dewi Subramaniam, Khadijah Husna Abd Hamid, Maria Pilar Almajano

**Affiliations:** 1 Faculty of Chemical and Process Engineering Technology, Universiti Malaysia Pahang, Lebuhraya Persiaran Tun Khalil Yaakob, Gambang 26300, Pahang, Malaysia; amninyahaya@gmail.com (W.A.W.Y.);; 2 Centre for Research in Advanced Fluid and Processes, Lebuhraya Persiaran Tun Khalil Yaakob, Gambang 26300, Pahang, Malaysia; 3 Chemical Engineering Department (DEQ), Escola Tècnica Superior d’Enginyeria Industrial de Barcelona (ETSEIB), Universitat Politècnica de Catalunya (UPC), Av, Diagonal 647, 08028 Barcelona, Spain

**Keywords:** seaweed derivatives, plasticizers, cellulose nanofibers, biopolymer, Gaussian

## Abstract

Biodegradable films made from biopolymer materials have the potential to replace conventional plastics, which can reduce waste disposal problems. This study aims to explore the potential of different seaweed derivate films consisting of 2% (*w*/*w*) of kappaphycus alverezi (KA), kappa carrageenan (KC), refined carrageenan (RC) and semi-refined carrageenan (SRC) as bio-based materials with 0.9% (*w*/*w*) glycerol (G), and reinforced with different concentrations of cellulose nanofibers (CNFs) derived from palm waste. A characterization of the glycerol-plasticized seaweed derivatives containing 0, 5, 10, and 15% (*v*/*w*) cellulose nanofiber is carried out. The CNFs were studied based on their mechanical, physical and thermal properties including mechanical properties, thickness, moisture content, opacity, water solubility, water vapor permeability and thermal stability. The hydrogen bonding was determined using the DFT calculation generated by Gauss view software version 9.6. The KA + G + 10%CNF film exhibited a surface with slight cracks, roughness, and larger lumps and dents, resulting in inferior mechanical properties (18.50 Mpa), making it unsuitable for biofilm production. The KC + G + 10%CNF film exhibited mechanical properties 24.97 Mpa and water vapor permeability of 1.42311 × 10^−11^ g s^−1^ m^−1^ Pa^−1^. The RC/G/10%CNF film displayed the highest TS (48.23 MPa) and water vapor permeability (1.4168 × 10^−11^ g s^−1^ m^−1^ Pa^−1^), but it also had higher solubility in water (66%). In contrast, the SRC + G + 10%CNF film demonstrated excellent mechanical properties (45.98 MPa), low water solubility (42.59%), low water vapor permeability (1.3719 × 10^−11^ g s^−1^ m^−1^ Pa^−1^), and a high decomposition temperature (250.62 °C) compared to KA, KC and RC. These attributes develop films suitable for various applications, including food packaging with enhanced properties and stability.

## 1. Introduction

Petroleum-based polymers such as polyethylene, polypropylene, and polystyrene, are widely used in the manufacture of commercial packaging materials such as plastic bags, bottles, and food containers [1]. Non-degradable materials often end up in landfills, oceans and other natural environments. It can take hundreds or even thousands of years for these materials to degrade, resulting in continued pollution of the environment and harm to wildlife. Therefore, biomass-based packaging materials derived from renewable resources, agricultural wastes, and agro-industrial byproducts have received attention due to their advantages such as low price, easy availability, and environmental friendliness compared to synthetic products [2]. Of the biodegradable polymers, polysaccharides derived from natural monomers offer the greatest opportunity to become alternative packaging materials due to their potential biodegradability and environmental compatibility [3]. Among biopolymers, seaweed-based biopolymers such as alginate, agar, and carrageenan have received great interest due to their good barrier properties and mechanical properties. Seaweed derivatives are mostly used in the food industry as thickeners, gelling agents and stabilizers in concentrations ranging from 0.005% to 2.0% (*w*/*w*).

Seaweed derivatives, such as Kappaphycus alvarezii (KA), kappa carrageenan (KC), refined-carrageenan (RC), and semi-refined-carrageenan (SRC), are used as biopolymers in the production of biodegradable films. These seaweeds are known for their high content of carrageenan, a polysaccharide that forms a gel-like substance when dissolved in water. KA, also known by the trade name cotonii, is a class of Rhodophyceae [4]. This species of algae occurs in reddish, yellowish and green colors, depending on the concentration of phycoerythrin pigment. It is easy to cultivate and grows rapidly, with an increase of almost 4.5% daily. KA is the main industrial source of κ-carrageenan [4,5], as this polysaccharide accounts for up to 37 wt% of the alga, on a dry weight basis. Due to the properties of κ-carrageenan, such as thickening, gelling, stabilization and emulsification, it has a variety of applications, such as a thickening agent for milk-based desserts. In general, the KA consists on average of 50.8% carbohydrates, 3.3% lipids, 3.3% proteins, 12.4% sulfated groups, 15.6% ash and 3.0% insoluble aromatics [6,7]. Meanwhile, semi-refined carrageenan (SRC) is the end product of KC processing, which involves alkali treatment to remove the carrageenan. Remaining components, such as cellulosic materials, can be removed using additional processes such filtration and purification, resulting in refined carrageenan (RC) [8]. SRC’s global pricing is often only two-thirds that of traditional RC because there are fewer processing stages involved. The cosmetics and pharmaceutical industries employ refined carrageenan extensively, whereas SRC is mostly used in other applications, such food packaging, that do not require high refinement. However, they face some problems due to their natural hydrophilicity, which frequently needs to be modified by grafting/blending with other polymers or by adding fillers to improve their competitiveness with standard polymers [9,10].

Oil palm biomass (OPB), which is made up of empty fruit bunches (EFBs), was abundantly generated in Malaysia and can be used as a source of cellulose for the production of nanocellulose where only 10% is used, and it is frequently left inexhaustible. These OPBs offer enormous resources for the translation into value-added products based on scientific results [11]. Nanocellulose is produced by breaking down cellulose fibers into nano-sized particles, which can be used to reinforce polymer composites and improve their mechanical properties. The extraction process of nanocellulose involves the removal of hemicellulose and lignin from wood pulp, followed by mechanical or chemical treatment to obtain the desired particle size (<100 nm). Dai et al. [12] has demonstrated the flexibility of hydroxypropyl guar/cellulose-nanofibrils films using a casting technique. In comparison to the control film, the composite films have better mechanical properties and higher oxygen and water vapor barriers. The manufacturing technology of biodegradable films involves mixing the biopolymers with other components, such as glycerol and nanocellulose, to form a composite material. The composite is then molded into thin films using techniques such as extrusion or casting. The economic market value of biodegradable films made from seaweed derivatives and nanocellulose is growing rapidly, as consumers and manufacturers become more aware of the environmental impact of conventional plastics. Grand View Research’s analysis estimates that the global market for biodegradable plastics was worth USD 3.39 billion in 2020 and is projected to increase at a CAGR of 14.5% from 2021 to 2028.

The increasing demand for sustainable packaging solutions, particularly in the food and beverage industry, is driving the growth of the biodegradable plastics market. Therefore, further studies were conducted by adding nano-fillers as reinforcing agents to meet the requirements of packaging applications. This study aims to explore the potential of these four comparison materials from seaweed derivatives such as KA (pure seaweed without the extraction process), KC (pure carrageenan), RC and SRC with natural plasticizers from glycerol, reinforced with different concentrations of cellulose nanofibers (0,5,10 and 15% *v*/*v*). The films produced were characterized based on mechanical, physical, and thermal properties and its morphology to develop a potential bio-nanocomposite film. Overall, this work has significant implications for the development of sustainable materials that can help reduce plastic waste and mitigate its environmental impact.

## 2. Materials and Methods

### 2.1. Materials

KA, KC, RC and SRC range < 200 µm powder size were obtained from TACARA Sdn., Bhd., and cellulose nanofiber (CNF) was purchased from UPM Biomass Centre, Malaysia. Glycerol (99%), hydrochloric acid (HCl), potassium chloride (KCl), and potassium oxide (KOH) were supplied by Sigma-Aldrich, Gillingham, England.

### 2.2. Preparation of Kappaphycus Alvarezii (KA)

The dried seaweed was pre-treated by removing visible foreign matters such as sand and stones. The seaweeds were further washed with running deionized water for 5 min to reduce salt content. Then, the preheated seaweed was dried in an oven at 60 °C until a constant weight, to fully remove the moisture content. The preheated seaweeds were grounded for 400 rpm using a Retsch ball mill grinder and sieved as powder <200 µm.

### 2.3. Extraction of κappa Carrageenan (KC)

The extraction of κappa-carrageenan was prepared according to Manuhara et al. [13]. The dried seaweed was washed with tap water and soaked in water for 24 h. After soaking, it were cut and crushed with a blender to prepare an algal slurry. Then the slurry was mixed with water and conditioned in an alkaline solution, which was heated at 90 °C for 2 h with constant stirring. After extraction, the residue was separated from the viscous filtrate. It was coagulated with a KCl solution for 15 min and stirred, and then the mixture was filtered to separate the water and carrageenan gel. The gel was completely soaked in 96% alcohol for one hour with continuous stirring. It was separated from the alcohol and water by filtration. Finally, it was dried at 70 °C for 24 h and ground into powder.

### 2.4. Extraction of Refined Carrageenan (RC)

Kappaphycus alvarezii was submerged in 3 L of water for 24 h after being rinsed with flowing water. Then, the algae were cut with scissors and ground with a blender to produce the algal pulp. The water and pulp were then combined in a ratio of 1:80 (*v*/*v*). Ca(OH)_2_ solution was used to condition the mixture into an alkaline state (pH 9). Then, the extraction was carried out by heating at 90 °C for two hours while stirring continuously. Following extraction, solid algal waste was isolated from the filtrate. The filtrate was then warmed at 60 °C for 30 min after being neutralized with a 1% HCl solution to a pH of 7. The mixture was filtered to separate the carrageenan gel and water after the filtrate had been coagulated with KCl solution (1.5%, 2.5%, or 3.5%) and KCl solution in a 1:1 ratio for 15 min. After that, the carrageenan gel was thoroughly dissolved in 96% alcohol and swirled constantly for an hour. Filtration was used to separate the carrageenan gel from the alcohol and water. The carrageenan was milled into an 80-mesh size after being dried in a cabinet drier at 70 °C for 24 h [6].

### 2.5. Extraction of Semi-Refined Carregeenan (SRC)

Semi-refined carrageenan was prepared according to the Normah and Nazarifah method [14], with slight modifications. *Kappaphycus alvarezii* were washed under running tap water to remove residues, sand and salt. Then, 200 g of algae were extracted in 2.5 L of hot alkaline solution containing about 150 g of potassium hydroxide solution with an alkaline pH of 13 at a temperature of 70 °C for 2 h. Then, the algae were neutralized by soaking in water for 4 h followed by overnight soaking. The neutralized algae were dried overnight at a temperature of 50 °C. Finally, the dried SRC were ground and stored in a desiccator for further analysis.

### 2.6. Preparation of Bio-Nanocomposite Films

The seaweed derivative powder was mix in distilled water (2% *w*/*w*, based on the dry weight) and heated to a temperature of 80 °C with constant stirring. Then, the plasticizer glycerol (0.9% *v*/*v*) was included at 80 °C under magnetic stirring. The temperature was maintained at 800 rpm for 10 min with continuous stirring to achieve gelation. After the addition of glycerol, the film solution was heated to 90 °C and the temperature of the film solution was maintained at 90 °C for ± 1 min. Then, the cellulose suspension was added at different concentrations (0, 5, 10, and 15% *v*/*v*). The control film was prepared without the addition of plasticizers. A total of 100 mL of the film-forming solution (FFS) was poured onto a polyacrylic casting plate (16 cm × 16 cm × 0.3 cm) and dried for 1 day at a temperature of 40 ± 2 °C. The dried film was eventually removed from the plates. Table 1 shows the different concentrations of the film sample.

### 2.7. Tensile Strength (TS) and Elongation at Break (EAB)

The TS and EAB of the films were evaluated using a testing machine (AG-Xplus Series, Shimadzu, Japan). Film samples were cut into 1.5 cm × 10 cm. Then, the samples were tested using the testing machine and measured with a deformation rate of 50 mm/min [15]. The tested film were equilibrated in desiccators at 25 °C and 50% relative humidity for 48 h. The TS and EAB of the film samples were measured according to the standard method ASTM D882-12. The TS and EAB value of the films were calculated using the following equation:

(1) $$\mathrm{Ts}\left(\mathrm{Mpa}\right)=\frac{F_{\max}}{\emptyset }$$

where F_max_ is the maximum load and Φ is the cross-sectional area of the film. The EAB of the films will be calculated using the following equation:

(2) $$\mathrm{EAB}\left(\%\right)=\frac{\Delta l}{l_{o}}\times 100$$

where ∆l is the film extension and l_0_ is the initial length of the film sample.

### 2.8. Thickness Measurement

A hand-held digital micrometer (Mitutoyo Co., Tokyo, Japan) was used to measure the thickness of the film samples. The mean values of five measurements were calculated at random positions of the film samples. The mean value of thickness was used for the opacity calculation and mechanical properties [16].

### 2.9. Opacity Measurement

The opacity of the film was measured by using a spectrophotometer. The film was cut into 3 cm × 0.3 cm and put into the cuvette, according to the ASTM D523-08 method proposed by Shojaee-Aliabadi et al. [17] The opacity was examined at 600 nm using UV–Vis. The opacity was calculated based on the following equation:

(3) $$Opacity=\frac{Abs600}{b}$$

where *Abs* 600 is the value of absorbance at 600 nm and *b* is the thickness of film (mm).

### 2.10. Solubility in Water

The water solubility (WS) of the film samples was evaluated using the Farhan and Hani method. The film samples were sliced uniformly (2 cm × 2 cm) and dried in a laboratory oven at 100 °C for 24 h. To ascertain their initial dry weight, they were weighed to the closest 0.0001 g. Then, the films were placed in 50 mL screw-capped centrifuge tubes with 30 mL of distilled water and put into a water bath with continuous stirring at 25 °C for 24 h. Undissolved films were then filtered using Whatman No. 1 filter paper and dried at 100 °C for 24 h to ascertain their final dry weight. Each sample was measured three times. The WS (%) was performed as a percentage, using the following equation:

(4) $$\mathrm{WS}\left(\%\right)=\frac{W_{O}-W_{f}}{W_{O}}\times 100$$

where W_O_ is the initial dried weight of the film and W_f_ is the final weight of the dried, undissolved film.

### 2.11. Moisture Content

The moisture content (MC) of the films was measured according to the method studied by Nur Fatin Nazurah and Nur Hanani [18]. The moisture contents of the film samples were determined by measuring the weight loss of the films (2 cm × 2 cm) before and after drying in a laboratory oven at 100 °C for 24 h. The measurement was performed in triplicate. MC was examined in percentage according to the equation below:

(5) $$\mathrm{MC}\left(\%\right)=\frac{{\mathrm{MC}}_{\mathrm{wet}}-{\mathrm{MC}}_{\mathrm{dry}}}{{\mathrm{MC}}_{\mathrm{wet}}}\times 100$$

where W is the weight of the film sample.

### 2.12. FT-IR Spectroscopy

Interactions of the different constituents within the *Kappaphycus alvarezii* seaweed, kappa-carrageenan, refined carrageenan and semi-refined carrageenan-based films incorporated with 10% of CNF film were obtained using a Fourier-transform infrared spectrometer (Thermo Scientific Nicolet iS5 FT-IR Spectrometer, Massachusetts, USA). The IR spectrum was measured over the wave number range from 4000 to 650 cm^−1^, using OMNIC software.

### 2.13. Thermal Properties

The TA Q500 thermogravimetric analyzer was used to analyze the thermal stability of the samples using the TGA method. A continuous heating rate of 10 °C per minute was used for the measurements. Under a nitrogen atmosphere, the temperature was varied from 30 °C to 600 °C [19]. The derivation of the TGA curves was used to calculate the decomposition temperatures (DTG).

### 2.14. Scanning Electron Microscopy

A small layer of gold was applied to the film specimens before they were placed on aluminum stubs with double-sided tape. A scanning electron microscope (Jeol, model JSM-5800, Tokyo, Japan) operating at 5 kV and a magnification of 1000 kx was used for morphological observations on the surface of the films (which were broken under liquid nitrogen before imaging).

### 2.15. Water Vapor Permeability

According to Nur Hanani, Roos, and Kerry (2012), the film sample was firmly placed over the cup’s rim and each crucible was filled with 6 mL of distilled water. To prevent leaks, a vacuum seal grease was employed. The crucibles were put into a desiccator with silica gel beads as a desiccant (50 ± 5% RH and 23 ± 2 °C). Over the course of eight hours, the weight differences were tracked at one-hour intervals, and the WVP was determined using the following [20]:

(6) $$\mathrm{WVP}=\frac{\Delta w.l}{A.t.\;P}$$

where Δw is the weight difference (g), l is the film thickness (m), A is the exposed area of the film (m^2^), t is the time elapsed, and P is the partial pressure difference of water vapor across the film (Pa).

### 2.16. Quantum Mechanic Simulation

The chemical interaction between carrageenan, glycerol, and cellulose nano-fiber was simulated using Gaussian 09W molecular dynamics software. The Pubchem database was used to obtain the molecular structures. To save costs on computation and simulation time, the chemical structures of carrageenan and cellulose nanofiber were shortened. The carrageenan structure was reduced to a single molecular unit, whereas the cellulose nanofiber structure was reduced to two units. DFT calculations were used to optimize the geometries of all molecules using Becke’s three parameters in conjunction with the correlation functions of Lee, Yang, and Parr (B3LYP) with a 6-31G (d,p) basis set. The molecular electronic surface potentials (MESPs) of the carrageenan, glycerol, and cellulose nanofibers were calculated using geometry optimization. The interaction energy was calculated using the generated energy of the self-consistent field (SCF), as shown below:

(7) $$Interaction\;energy=E_{SCFcomplex}-(E_{SCFcarrageenan\;}+E_{SCFglycerol\;}+E_{SCFcnf})$$

## 3. Results

### 3.1. Mechanical Properties of Films

TS, EAB and Young’s modulus are commonly used to determine the breaking strength of the packaging materials. The value of TS indicates the resistance of the film at maximum tensile load, while the value of EAB (%) indicates the maximum allowable elongation of the film [21]. Meanwhile, Young’s modulus indicates the elasticity that characterizes the stiffness or flexibility of the material. Table 2 shows the TS (MPa), EAB (%) and young modulus value of all treated samples.

The addition of glycerol 0.9% as a plasticizer significantly changes the value of TS and EAB% for all samples (*p* < 0.05) compared to the unplasticized film, decreasing the brittleness of the sample and increasing the elasticity of the film. According to Balqis et al., these phenomena are due to the increased spatial distance between the polymer chains, located between the polymer molecules, due to the plasticizers [18]. On the other hand, EAB was found to be inversely correlated with TS. Plasticizers such as glycerol increase the mobility of the polymer chains, resulting in more stretchable and flexible films, which increases the EAB and decreases the TS. Further improvements were achieved by adding CNF as a filler. This led to positive results, as the mechanical properties of the film improved. This indicates that hydrogen bonds are formed between CNF and carrageenan, which increases the cross-linking between polymer chains and CNF fills the voids created by plasticizers, which eventually decreases the movement of chains. This finding was supported by Bagheri et al. (2019) in their studies, where the addition of CNF to whey gluten improved its mechanical properties [22]. On the other hand, for kappa-carrageenan (KC) and semi-refined carrageenan (SRC), the TS decreases after the addition of 15% CNF. This is due to the inhomogeneity of the biopolymers and the CNF composite film, where the CNF agglomerates and is incompatible with the biopolymer matrix. The agglomeration of the fibers affects the result, because the structure of the polymer chains is different [23]. According to Zare et al. 2016, agglomeration is attributed to direct mutual attraction between nanoparticles through van der Waals forces or chemical bonding [24]. A similar finding was observed by Sogut et al., in that the aggregation potential of CNF particles occurs once a sufficient concentration is reached in the film solution [25]. Apart from this, Xu et al. observed that CNFs reinforced with chitosan improved the mechanical properties of the film from 2% to 10%, resulting in a significant increase in elongation at the break, due to better adhesion of the nanosized cellulose to the matrix of the biopolymers [26]. Meanwhile, KA with 15% CNF increased in TS but with a lower EAB compared to other samples, due to its properties which make the KA film easy to break and prevent it forming good mechanical properties. Biopolymer films with a suitable plasticizer concentration can be obtained with good mechanical properties in the range of 10–100 Mpa. Moreover, the reinforced CNF film is in the range of 10–100 Mpa, which shows good mechanical characteristics that can be used for the film development [27]. In addition, the modulus of elasticity provides valuable information about the ability of the films to resist external forces and maintain their structural integrity. A higher modulus of elasticity indicates a stiffer material with lower elasticity, while a lower modulus of elasticity indicates a more flexible and elastic film [28]. Among the control samples, KA, KC, RC and SRC, SRC had a relatively high Initial Young’s modulus of 49.83. However, with the addition of glycerol, the Young’s modulus decreased significantly to 2.71, 0.82, 2.20 and 2.28, respectively, indicating increased flexibility. The addition of CNF to KA + G films had different effects on the elastic modulus. While KA + 5%CNF slightly increased the elastic modulus to 2.82, KA + 10%G and KA + 15%G decreased it to 2.81 and 2.15, respectively, indicating increased flexibility with increasing CNF content. In contrast, KC-reinforced CNF was affected by the further addition of 5%, 10%,and15% CNF, remaining low at 1.19, 1.64, and 1.64, respectively. The elastic modulus of RC reinforced with 5%, 10%,and 15% CNF resulted in higher values of elasticity. The highest initial elastic modulus was found for the SRC samples (49.83), while SRC + G films was slightly decreased by the addition of CNF, indicating higher flexibility.

### 3.2. Physical Properties of Films

The thickness of packaging films is one of the most important factors in product protection. The gas permeability of the film can be affected by the thickness of the film. As the thickness increases, the gas permeability of the film can also increase. Table 3 shows the effect of glycerol and different concentrations of cellulose nanofibers (CNF) on the thickness of seaweed derivative films.

The thickness of all biopolymer film increased significantly after the addition of glycerol and CNF (*p* > 0.05), except for Kappaphycus Alvarezi (KA). This is due to the plasticizer molecules in the film matrix increasing the interstitial space between the polymer chains in the film matrix, resulting in an increase in thickness [16]. According to Ili Balqis et al. (2017), the thickness of the film increased because glycerol tends to absorb more moisture than an unplasticized film. Therefore, these films swelled to a greater extent, increasing their thickness. In addition, the thickness increased when CNF was added, as the solid content of the resulting film increased, with CNF serving as a filler for the space created by the plasticizers [29].

The film transparencies are shown in Table 3. The lower opacity values determined good film transparency, making its properties more attractive and clearer. The KC/G control film shows the highest transparency value, while the SRC control shows the highest opacity value. For all four types of seaweed derivative films, the initial opacity value is higher (without plasticizer), but the opacity value decreases when glycerol is added, and it increases when CNF is added, depending on the concentration. According to Farhan et al., the presence of glycerol reduces the intermolecular interactions between the polymer chains while increasing the space between them, allowing light transmission into the biopolymer film and resulting in great transparency. Because of the strong contact between CNF and the biopolymer matrix, CNF functions as a filler in the plasticized biopolymer matrix, resulting in lesser light scattering and increased light transmittance in the films. [2]. Meanwhile, SRC has the highest value for opacity, which is due to the presence of the cellulose that was present in the original algae. Therefore, it gives a turbid solution compared to RC and KC, which give a clear solution, while KA (the original alga) gives a clear solution compared to SRC, due to some impurities in the SRC derivatives [30].

Film solubility serves as an indicator of the quality of films used as packaging materials, including their integrity, water resistance, and biodegradability. In certain applications, it may be necessary for films to be water-insoluble in order to enhance product integrity and water resistance. As demonstrated in Table 3, adding plasticizers and CNF considerably increased the films’ solubility (*p* > 0.05). The result shows that the enhancement of CNF in the seaweed-based film significantly decreased the film solubility compared to the control films, which almost completely dissolved during the analysis as a result of the hydrophilicity of carrageenan. Film solubility increased when a plasticizer was introduced into the biopolymer matrix, compared to the unplasticized film. This is due to the nature of the plasticizer itself, which has hydrophilic properties [16,29]. Previous studies have shown that in the case of CNF, the primary factor contributing to a decrease in water solubility is the filling of voids between biopolymer chains by CNF, which reduces the mobility of these chains and consequently slows down the diffusion rate of water molecules [22]. Cellulose nanofibers (CNFs) improve the compactness of the biopolymer structure and increase resistance to water molecule permeability. This implies a stronger interaction between the biopolymer chains and CNFs, which is facilitated by better dispersion of the nanoparticles within the polymer matrix [31]. Another explanation for the decrease in water solubility with increasing CNF concentration, as illustrated in morphology image, could be the large molecular size and low solubility of CNFs in water [2].

Moisture content in the films can be measured by determining the percentage of moisture loss. Table 3 indicates a significant increase in percent moisture content (MC) in film plasticized with glycerol as compared to control films (without plasticizer) (*p* < 0.05). The percent moisture content for all control films (non-plasticized) shows the lowest value of MC compared to the other samples. It shows a drastic change when glycerol is added, which could be due to the hydrophilic nature of glycerol, where a higher number of hydroxyl groups (OH) are present in the plasticizer, leading to an increase in MC (Ili Balqis et al., 2017) [32]. Increasing hydrophilic plasticizers’ concentration leads to a reorganization of the polysaccharide network, free volume, and an increase in segmental movements, which makes it easier for water molecules to diffuse and results in a greater moisture content of the film. [33].

### 3.3. FT-IR Spectroscopy

**Figure 1:**
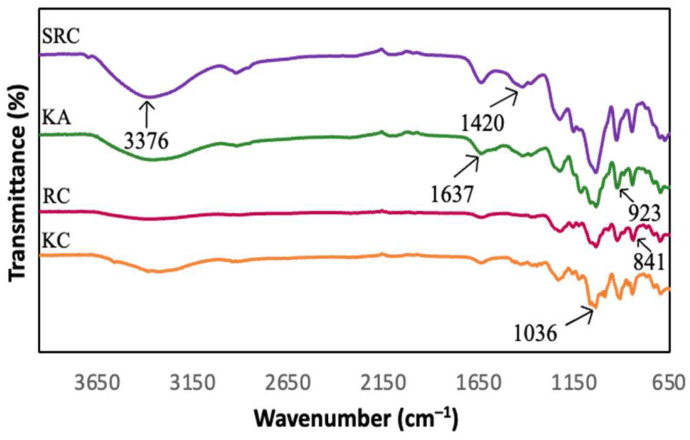
FT-IR spectra of different carrageenan-based films.

**Figure 2:**
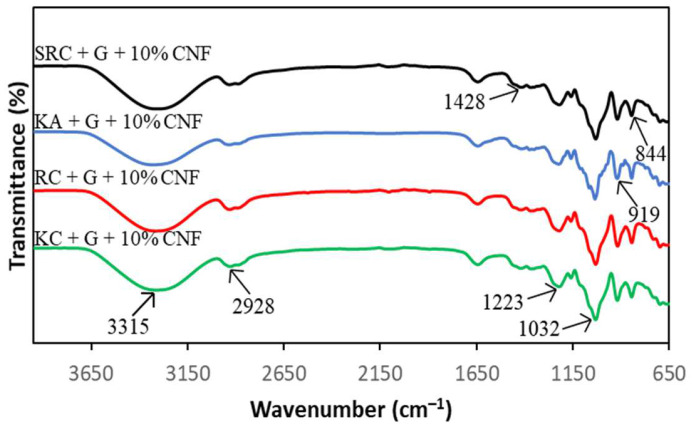
FT-IR spectra of different carrageenan-based films incorporated with 10% CNF.

FT-IR spectroscopy presented information about the chemical composition and specific functional groups of the carrageenan-based films for this study. Figure 1 and Figure 2 show the spectra of the absorption bands between 650 cm^−1^ and 3600 cm^−1^ for KA-, KC-, RC- and SRC-based films incorporated with 10% of CNF.

All films exhibited a broad absorption band at 3600 cm^−1^ to 3200 cm^−1^, due to the −OH stretching vibrations of carrageenan [34,35,36]. The neat KA, KC, RC and SRC films exhibit several absorption peaks at approximately 3376 cm^−1^, 1420 cm^−1^, 1637 cm^−1^, 1036 cm^−1^, 923 cm^−1^ and 841 cm^−1^ corresponding to the O–H stretching vibration, the C–H stretching vibration, the –OH stretching vibration, and the O–H bending vibration [37]. The absorption band at 1032 cm^−1^ was assigned to the glycosidic bond of the carrageenan group as described by Roy and Rhim [38]. The prominent peaks at 919 cm^−1^ and 844 cm^−1^ corresponded to 3,6-anhydrogalactose and galactose-4-sulphate, respectively, which referred to the kappa carrageenan [39]. The band spectra observed at 1223 cm^−1^ were attributed to the ester sulphate (S=O) group of carrageenan [40]. In the case of CNF, the peak around 2928 cm^−1^ is due to the −CH stretching vibration of the alkane group of the cellulose chain [41]. The band spectrum at 1428 cm^−1^ corresponding to O−C−H and H−C−H deformation was observed [42]. However, there were no significant changes in the position of absorption bands for all the films with various types of carrageenan incorporated with CNF.

### 3.4. Thermal Stability

**Figure 3:**
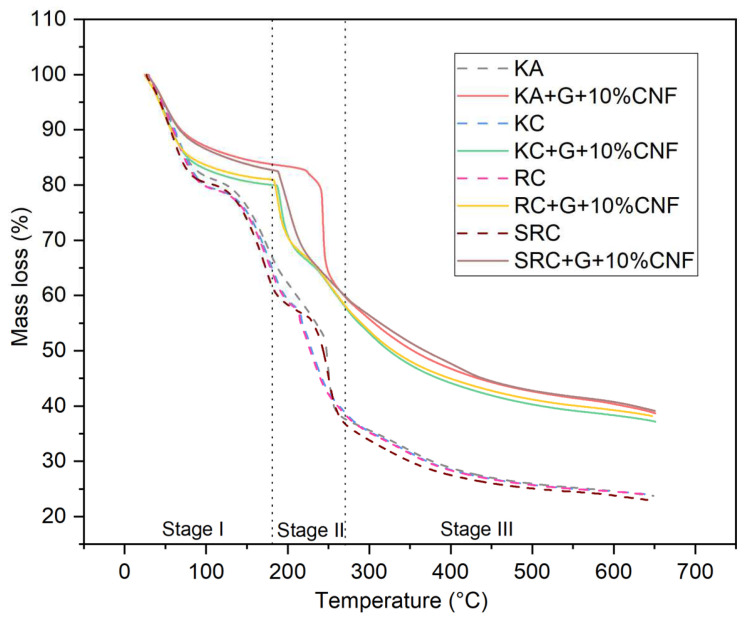
TGA of seaweed derivative bioplastic film matrices loaded with cellulose nanofiber.

TGA thermograms representing the thermal degradation behavior of biofilms with different biopolymer and cellulose nanofiber concentrations are presented in Figure 3.

**Figure 4:**
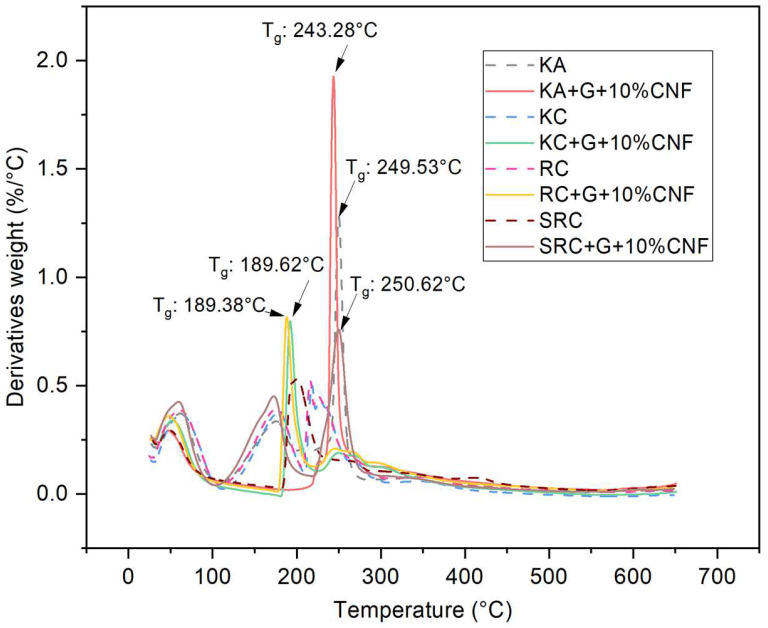
DTG of seaweed derivatives bioplastic film matrices loaded with cellulose nanofiber.

All biofilms experienced a three-stage mass loss at different temperature: 60–200 °C (weight loss: 16.42–20.77%), in the range of 210–290 °C (weight loss: 15.70–42.24%), and above 290 °C (weight loss: 15.91–33.93%). This can be explained by the evaporation of moisture followed by the volatilization of glycerol and the subsequent decomposition of the CNF polysaccharide [43,44]. In the first phase, which occurs at temperatures above 100 °C, the mass loss is due to the release of water molecules from the OH groups of the polysaccharide chains. CNF did not undergo significant thermal degradation in this phase, and the mass loss was mainly caused by water evaporation in the film sample. The predominant mass loss is represented by KA, RC and SRC at 100 °C with a weight loss of 18.963, 17.27 and 16.42%, respectively, due to water loss [45]. The second phase of weight loss was exhibited at 210–290 °C, with a weight loss of 15.70–42.24% which was related to the degradation caused by the loss of glycerol and water-binding structure. The addition of glycerol resulted in an increase in decomposition temperature, indicating that the polymer was partially stabilized by the plasticizer. The third stage, which occurs at temperatures above 290 °C, is attributed to the breakdown of the intramolecular and intermolecular hydrogen bonds of the cellulose nanofiber structure [46]. In contrast, the highest mass loss temperature of the biopolymer film tended to increase when cellulose nanofiber was added. This demonstrated the increased thermal properties of the film containing cellulose nanofiber with different seaweed derivative bio-based films. According to Viera 2018, at temperatures above ~290 °C, the degradation mechanism was considered as a fast degradation reaction which was associated with the destruction of hydrogen bonds leading to changes in crystallinity as well as the formation of free radicals, carbonyl, and carboxyl groups that accelerate primary cellulose degradation. The temperature at which the highest mass loss rate occurs is commonly defined as the decomposition temperature (Td), and is clearly visible as a peak in the DTG thermogram which reports the mass loss rate and temperature as shown in Figure 4.

The T_d_ of the biofilm-reinforced cellulose nanofiber was observed at 189–250 °C. The difference in the seaweed derivatives present in the biofilm may explain the changes in the Td value of the active film. SRC + G + 10% CNF alone has a higher T_d_ (∼210 °C) compared with other films, which might possibly be due to the higher energy for decomposition, because it exhibited good compatibility between SRC and CNF. These results support esthose of Moura et al. (2009), according to which the Td of hydroxypropyl methylcellulose (HPMC) is increased by the addition of chitosan-TPP nanoparticles, due to the strong H-bonding between HPMC and the nanoparticles [47]. A higher crystallinity of CNF leads to the higher enthalpy (energy) that is required to dissolve the crystalline structure, which improves the thermal stability of the material. Therefore, the degradation temperatures of the active films were increased compared to the control film. A similar study was described by Soni et al., in which the incorporation of cellulose nanofibers improved the thermal properties of the active film [48].

### 3.5. Morphology of Film

**Figure 5:**
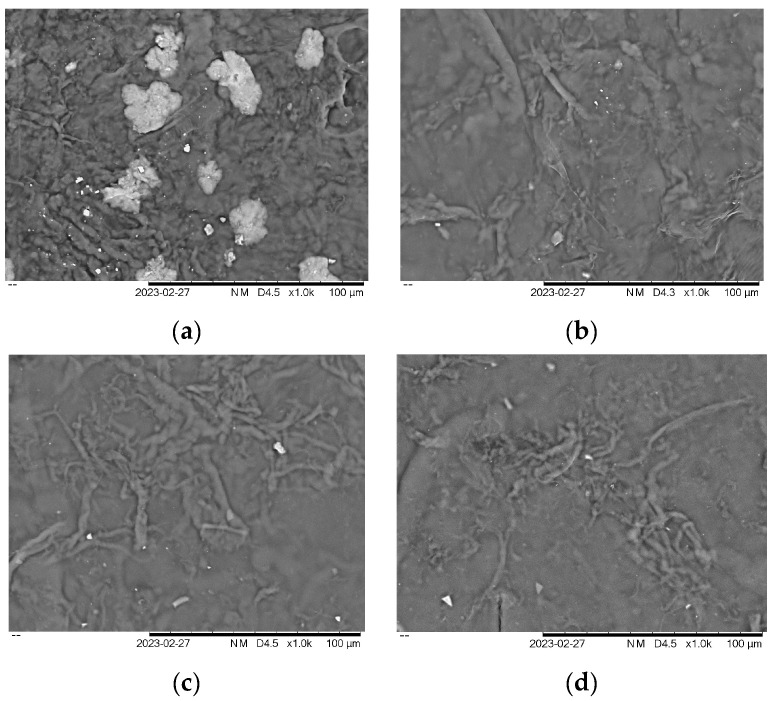
Morphology of film (**a**) KA + G + 10%CNF (**b**) KC + G + 10%CNF (**c**) RC + G + 10%CNF (**d**) SRC + G + 10%CNF.

SEM was used to examine the surface morphological features of the seaweed derivatives and cellulose nanofiber matrices, revealing the detailed surface structure of the film samples such as cracks, roughness, and nanoparticle distribution. Four selected samples were chosen to investigate their morphology based on mechanical and physical properties. The morphology of carrageenan films can vary, depending on the specific type of seaweed derivatives and the processing conditions used to prepare the film. As shown in Figure 5a, the surface of KA + G + 10%CNF appeared to be slightly cracked, rougher, and with larger lumps and dents compared to Figure 5b–d.

This surface is mainly due to the addition, which could be due to the polysaccharides present in the seaweed. This corroborates with the mechanical properties of the KA + G + 10%CNF film, which has low mechanical properties, and the uneven physical properties of the film which are not suitable for producing biofilm. The addition of nanoparticles changed the surface roughness of all the biopolymer films. Surfaces loaded with cellulose nanofiber in seaweed derivative biopolymer film matrices showed irregular bumps or ridges, which could contribute to air trapping and make the surface hydrophobic. Biopolymer KC, RC, and SRC, on the other hand, appeared even more smooth and wavy, indicating superior compatibility with the film matrix. The increase in surface roughness and surface morphology seen after the addition of nanoparticles was consistent with previous studies [49,50,51]. Similarly, the inclusion of ZnO and CuO nanoparticles resulted in a rougher and less homogeneous surface, due to the close packing/agglomeration of nanoparticles [52]. The distribution of CNF suspension inside the matrix, which creates a strong interaction with the film, influenced the improvement in bio-nanocomposite film characteristics.

### 3.6. Water Vapor Permeability

**Figure 6:**
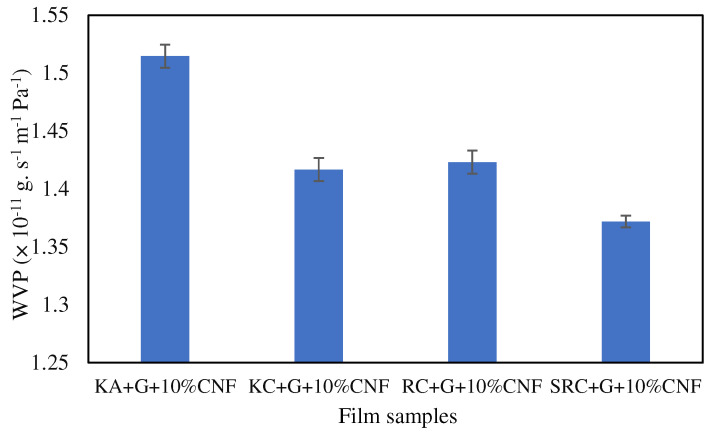
Water vapor permeability of biofilm.

Water vapor permeability (WVP) refers to the ability of a material to allow water vapor to pass through it. The WVPs of the KA-, KC-, RC- and SRC-reinforced 10% CNF bio-nanocomposite films are shown in Figure 6.

Lower values of water vapor permeability imply that the films offer better resistance to water vapor transmission. The film sample designated as SRC + G + 10%CNF (1.3719 × 10^−11^ g s^−1^ m^−1^ Pa^−1^) has the lowest water vapor permeability value, indicating that it provides the highest barrier to water vapor permeability. On the other hand, the film sample KA + G + 10%CNF (1.5146 × 10^−11^ g s^−1^ m^−1^ Pa^−1^) has the highest value for water vapor permeability, indicating that it provides the lowest resistance to water vapor permeability among the given samples. Meanwhile, the WVP values of RC + G + 10%CNF and KC + G + 10%CNF show no significant differences, with 1.4168 × 10^−11^ g s^−1^ m^−1^ Pa^−1^ and 1.42311 × 10^−11^ g s^−1^ m^−1^ Pa^−1^, respectively. This WVP value is smaller than the value observed by Oun and Rhim (1.44 × 10^−9^ g m/m^2^ s Pa), where crystalline 10% CNF was reinforced with sodium carboxymethyl cellulose (CMC) film [53]. The impermeable CNF, which was evenly dispersed throughout the polymer matrix, provides a convoluted path for water vapor diffusion and enhances the effective diffusion path length, causing a decrease in the WVP value of the composite film. This outcome is in line with previous research on polymer blends used in packaging [54,55]. The addition of cellulose nanocrystal CNC in CMC/ST blends had a positive effect on the WVP of the resulting bio-nanocomposite films. The WVP of the bio-nanocomposite films decreased to 4.22 × 10^−7^, 4.01 × 10^−7^, and 5.24 × 10^−7^ g m/m^2^ h Pa when 0.5, 2.5, and 5.0 wt% CNC were added, respectively [54]. Moreover, previous studies reported that SRC alone exhibited a high WVP of 10.47 g m/m^2^ h Pa compared to SRC-reinforced nanofiller [28]. This is due to the chemical structure of polysaccharides, which have a hydrophilic character unless modified and reinforced or plasticized with fillers [16]. In comparison, common plastics such as LDPE (low-density polyethylene), HDPE (high-density polyethylene), and WDC (water-resistant disposable coating) are known for their superior water vapor permeability (WVP), with values less than 0.1 gmm.m^−2^ d^−1^ kPa^−1^, exhibiting excellent moisture barrier properties, and making them a common choice for packaging applications [27]. However, their non degradable properties have become a main concern because of their environmental impact.

### 3.7. Quantum Mechanic Simulation

**Figure 7:**
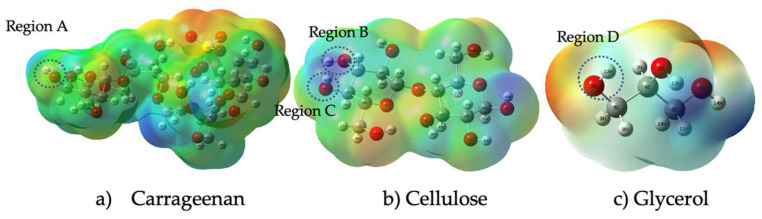
MESP structure of carrageenan, cellulose, and glycerol.

The distribution of charges and their respective intensities over the entire surface of these polysaccharides are shown in the optimized molecular electrostatic potential (MESP) of Figure 7.

**Figure 8:**
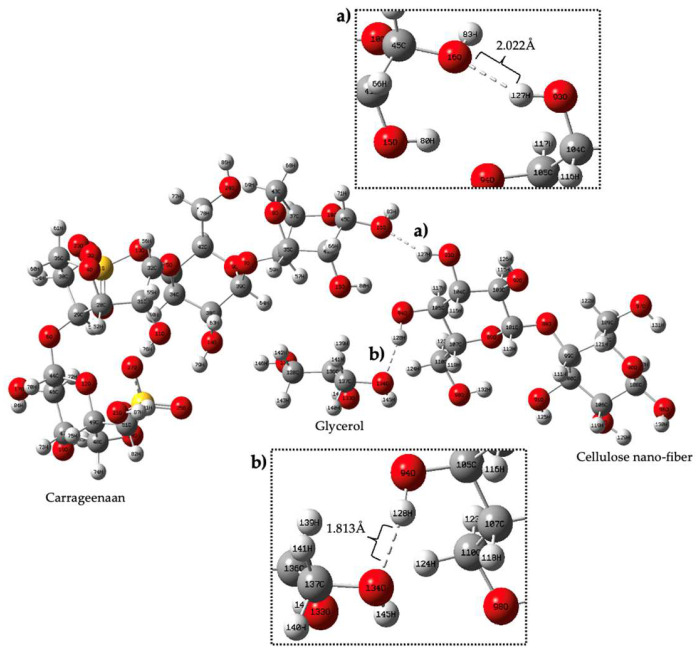
Complex structure of (**a**) conjugate carrageenan—cellulose nanofiber, (**b**) conjugate cellulose nanofiber—glycerol.

According to this figure, the electron potential increases with increasing electron density, with blue denoting the lowest electron density and red denoting the highest electron density. This indicates that the red-colored regions have a higher electron density than the blue-colored regions. It further states that, according to the Mulliken charge, a measure of partial charge distribution within a molecule, the oxygen atom in Region A of carrageenan has negative electrostatic potential and the hydrogen atom in Region B has positive electrostatic potential (yellow). Meanwhile, hydrogen bonding between Region C (atom H) and Region D (atom O) was determined based on their electrostatic potential blue color and yellow color, respectively. According to Kollman and Leland, a hydrogen bond is a type of chemical bond that occurs between a hydrogen atom and an electronegative atom such as oxygen, nitrogen, or fluorine in another molecule. It is an electrostatic attraction between a positively charged hydrogen atom (which is covalently bonded to one electronegative atom) and the lone pair of electrons on the electronegative atom of another molecule. The range of a hydrogen bond depends on a number of factors, including the strength of the bond and the specific atoms and molecules involved. In general, the distance between the hydrogen atom and the atom it is bonded to (typically nitrogen, oxygen, or fluorine) is typically between 1.5 and 2.5 angstroms (Å) [56]. In general, stronger hydrogen bonds tend to have shorter bond lengths and higher bond energies, while weaker hydrogen bonds have longer bond lengths and lower bond energies. Figure 8 shows the formation of a hydrogen bond between three different functional groups, namely carrageenan, cellulose nanofiber and glycerol.

The bond is formed between the hydrogen atom of carrageenan and the cellulose nanofiber, with a distance of 2.02 Å (angstroms) between them. The bond is formed due to the difference in electron density between the two regions, where the Mulliken atomic charge of the oxygen atom in region A is −0.563113 and that of the hydrogen atom in region B is 0.33. Meanwhile, the Mulliken atomic charge between the glycerol and the cellulose nanofiber was −0.5529 and 0.3057, respectively. In addition, the interaction energy for each component, the carrageenan, cellulose nanofiber and glycerol is −9.12 × 10^6^ kJ/mol, −3.28 × 10^6^ kJ/mol and −8.71 × 10^5^ kJ/mol. The interaction energy of the hydrogen bond between those three compounds is −27.59 × 10^6^ kJ/mol. These values indicate the compatibility of the film conjugate through hydrogen bond formation, which contributes to the potential development of the biopolymer film. The high interaction energy values of the conjugate are attributed to the existence of a strong interaction with the intermolecular hydrogen bond.

## 4. Conclusions

In conclusion, the study revealed that different seaweed derivatives exhibited varying characteristics, highlighting their potential applications. The biopolymer films derived from seaweed derivatives generally demonstrated favorable mechanical properties, with tensile strength (TS) ranging between 10 and100 MPa and elongation at break (EAB) exceeding 10%, except for the KA biofilm. The incorporation of cellulose nanofibers (CNF) positively influenced the TS and EAB of the films, while also affecting their physical properties such as solubility, opacity, and moisture content. However, the KA + G + 10%CNF film exhibited a surface with slight cracks, roughness, and larger lumps and dents, resulting in inferior mechanical properties (18.50 Mpa) and an uneven physical structure, making it unsuitable for biofilm production. The KC + G + 10%CNF film exhibited mechanical properties of 24.97 Mpa and water vapor permeability of 1.42311 × 10^−11^ g s^−1^ m^−1^ Pa^−1^. On the other hand, the RC/G/10%CNF film displayed the highest TS (48.23 MPa), but it also had higher solubility in water (66%). In contrast, the SRC/G/10%CNF film demonstrated excellent mechanical properties (45.98 MPa), low water solubility (42.59%), low water vapor permeability (1.3719 × 10^−11^ g s^−1^ m^−1^ Pa^−1^), and a high decomposition temperature. These attributes make it a promising candidate for packaging films incorporating active agents. Overall, this research highlights the importance of selecting the appropriate seaweed derivative and optimizing the composition to achieve the desired film properties. By understanding the effects of CNF addition and the specific characteristics of different seaweed derivatives, it becomes possible to develop films suitable for various applications, including food packaging, with enhanced properties and stability.

## Figures and Tables

**Table 1:** The different concentrations of film sample.

Sample	Seaweed Derivatives	Glycerol	Cellulose Nanofiber
KA	KA	0	0
KA + G	KA	0.9	0
KA + G + 5%CNF	KA	0.9	5
KA + G + 10%CNF	KA	0.9	10
KA + G + 15%CNF	KA	0.9	15
KC	KC	0	0
KC + G	KC	0.9	0
KC + G + 5%CNF	KC	0.9	5
KC + G + 10%CNF	KC	0.9	10
KC + G + 15%CNF	KC	0.9	15
RC	RC	0	0
RC + G	RC	0.9	0
RC + G + 5%CNF	RC	0.9	5
RC + G + 10%CNF	RC	0.9	10
RC + G + 15%CNF	RC	0.9	15
SRC	SRC	0	0
SRC + G	SRC	0.9	0
SRC + G + 5%CNF	SRC	0.9	5
SRC + G + 10%CNF	SRC	0.9	10
SRC + G + 15%CNF	SRC	0.9	15

KA: kappaphacus Alvarezii KC: Kappa carrageenan RC: refined carrageenan SRC: semi-refined carrageenan.

**Table 2:** Result of mechanical strength of films.

Sample	Mechanical Properties		
	TS (Mpa)	EAB (%)	Young Modulus
KA	11.36 ± 1.22 ^a^	1.99 ± 1.03 ^a^	5.71
KA + G	15.02 ± 1.64 ^b^	5.54 ± 2.09 ^b^	2.71
KA + G + 5%CNF	16.79 ±0.86 ^c^	5.96 ± 1.64 ^c^	2.82
KA + G + 10%CNF	18.50 ± 0.53 ^d^	6.58 ± 1.12 ^d^	2.81
KA + G + 15%CNF	20.21 ± 1.08 ^e^	9.40 ± 1.39 ^e^	2.15
KC	46.87 ± 1.1 ^a^	0.97 ± 0.03 ^a^	48.23
KC + G	18.31 ± 0.04 ^b^	22.32 ± 0.13 ^b^	0.82
KC + G + 5%CNF	24.22 ±0.16 ^c^	20.27 ± 0.20 ^c^	1.19
KC + G + 10%CNF	24.97 ± 2.83 ^d^	15.22 ± 1.39 ^d^	1.64
KC + G + 15%CNF	22.08 ± 1.40 ^e^	13.45 ± 0.99 ^e^	1.64
RC	59.03 ± 0.48 ^a^	2.65 ± 0.13 ^a^	22.27
RC + G	40.63 ± 0.52 ^b^	18.46 ± 0.16 ^b^	2.20
RC + G + 5%CNF	42.90 ±6.98 ^c^	11.10 ± 1.73 ^c^	3.86
RC + G + 10%CNF	48.23 ± 2.54 ^d^	8.22 ± 1.68 ^d^	5.87
RC + G + 15%CNF	41.39 ± 7.34 ^e^	12.86 ± 2.71 ^e^	3.22
SRC	50.83 ± 1.52 ^a^	1.02 ± 0.13 ^a^	49.83
SRC + G	36.08 ± 1.79 ^b^	15.82 ± 1.06 ^b^	2.28
SRC + G + 5%CNF	39.63 ±0.95 ^c^	23.56 ± 3.88 ^c^	1.68
SRC + G + 10%CNF	45.98 ± 0.57 ^d^	19.18 ± 0.78 ^d^	2.39
SRC + G + 15%CNF	26.72 ± 2.28 ^e^	20.28 ± 3.42 ^e^	1.32

The mean and standard deviation of the values are shown. Differing letters in the same column denote substantially different values (*p* < 0.05).

**Table 3:** Result of physical properties of films.

Sample	Physical Properties			
	Thickness (mm)	Opacity	Water Solubility (%)	Moisture Content (%)
KA	0.060 ±0.00	4.50 ± 0.40 ^b^	60.00 ± 4.60 ^b^	1.64 ± 0.27 ^b^
KA + G	0.060 ±0.00	3.86 ± 0.26 ^b^	67.92 ± 0.30 ^c^	39.23 ± 0.33 ^c^
KA + G + 5%CNF	0.060 ± 0.00	5.63 ± 0.50 ^c^	57.01 ± 4.02 ^d^	30.18 ± 1.13 ^d^
KA + G + 10%CNF	0.060 ± 0.00	7.32 ± 0.25 ^c^	47.22 ± 3.48 ^e^	28.85 ± 0.12 ^d^
KA + G + 15%CNF	0.060 ± 0.00	7.29 ± 0.18 ^c^	43.92 ± 0.39 ^f^	27.31 ± 0.39 ^e^
KC	0.040 ± 0.00	9.98 ± 1.10 ^a^	60.52 ± 5.40 ^a^	1.98 ± 0.07 ^a^
KC + G	0.092 ± 0.01 ^b^	1.90 ± 0.12 ^b^	80.00 ± 6.72 ^a^	35.83 ± 0.16 ^b^
KC + G + 5%CNF	0.092 ± 0.01 ^b^	3.09 ± 0.25 ^b^	55.45 ± 1.36 ^b^	30.27 ± 1.05 ^c^
KC + G + 10%CNF	0.092 ± 0.01 ^c^	3.92 ± 0.03 ^c^	53.26 ± 0.66 ^c^	30.12 ± 0.08 ^d^
KC + G + 15%CNF	0.100 ± 0.00	3.93 ± 0.02 ^c^	53.01 ± 5.92 ^d^	29.62 ± 0.29 ^d^
RC	0.020 ± 0.00	3.65 ± 0.48 ^c^	82.00 ± 0.57 ^e^	8.48 ± 0.36 ^e^
RC + G	0.024 ± 0.01 ^c^	2.68 ± 0.30 ^c^	88.03 ± 7.89 ^f^	35.61 ± 0.16 ^e^
RC + G + 5%CNF	0.040 ± 0.00	3.51 ± 0.29 ^a^	68.18 ± 0.81 ^a^	29.08 ± 0.54 ^a^
RC + G + 10%CNF	0.040 ± 0.00	6.94 ± 1.38 ^b^	66.71 ± 3.68 ^a^	29.06 ± 0.95 ^b^
RC + G + 15%CNF	0.040 ± 0.00	7.27 ± 0.78 ^b^	66.00 ± 0.54 ^b^	26.24 ± 0.41 ^c^
SRC	0.068 ± 0.01 ^c^	10.88 ± 0.5 ^c^	66.69 ± 2.09 ^c^	1.50 ± 0.08 ^d^
SRC + G	0.072 ± 0.01 ^c^	7.91 ± 0.44 ^c^	93.19 ± 6.18 ^d^	31.85 ± 0.48 ^d^
SRC + G + 5%CNF	0.072 ± 0.01 ^c^	8.62 ± 0.02 ^c^	53.04 ± 2.59 ^e^	29.36 ± 0.39 ^e^
SRC + G + 10%CNF	0.080 ± 0.00	8.68 ± 0.17 ^c^	42.59 ± 7.78 ^a^	28.84 ± 0.71 ^e^
SRC + G + 15%CNF	0.088 ± 0.01 ^c^	8.85 ± 0.85 ^a^	40.00 ± 2.86 ^a^	27.35 ± 0.29 ^a^

The mean and standard deviation of the values are shown. Differing letters in the same column denote substantially different values (*p* < 0.05).

## Data Availability

Not applicable.
